# Self-reported safety practices and associated factors among employees of Dashen brewery share company, Gondar, Ethiopia: a cross-sectional study

**DOI:** 10.1186/s12995-017-0169-2

**Published:** 2017-08-04

**Authors:** Solomon Tesfa Tezera, Daniel Haile Chercos, Awrajaw Dessie

**Affiliations:** Department of Environmental and Occupational Health and Safety, Institute of Public Health, University of Gondar, P.O. Box 196, Gondar, Ethiopia

**Keywords:** Safety practice, Prevalence, Brewery factory, Ethiopia

## Abstract

**Background:**

According to International Labor Organization (ILO), occupational accidents and work-related diseases are the causes for millions of deaths of workers every year. In addition, many millions of workers suffer non-fatal injuries and illnesses. This research was conceived with aim to assess safety practices and associated factors among employees of Dashen brewery Share Company, Ethiopia.

**Method:**

Institutionalbased cross-sectional study was conducted to assess the level of self-reported safety practice and associated factors from February to March 2016, among Dashen brewery workers. Stratified sampling method was employed to select 415 study participants and the data was collected by using structured interview-administer questionnaire. Observational checklist was also used to ascertain the response given by interviewee.

**Results:**

Fourhundred 15 respondent were involved in this study. Of those individuals, almost three fourth (74.2%) of the participants were male and 43.4% of participants were single. Mean (SD) age of respondents were 28.18 (±8.67) years and half of the respondents (49.9%) were diploma holders. The finding of this study indicated that 87.2% of the respondents reported complying with good safety practice. Age, marital status, employment status, attitude, safety and health training, and management support were found to be main predictors for safety practices.

**Conclusion:**

The level of self-reported safety practice in this study was good. Management commitment on safety and training of the employees about safety and health is very important and should be provided regularly.

**Electronic supplementary material:**

The online version of this article (doi:10.1186/s12995-017-0169-2) contains supplementary material, which is available to authorized users.

## Background

Millions of industrial workers around the world are involved in different hazardous work-related exposures on a daily basis. Due to the presence of hazards, employees in both developed and developing countries are highly vulnerable for diverse and considerable risk of industrial accidents, diseases and death [1].

According to the International Labor Organization (ILO), an estimated 2.3 million workers die every year from occupational accidents and work-related diseases globally. The total economic loss due to this is tremendous [2]. The majority of labor force in developing countries live and work in hazardous work environment that worsens their health, social and economic condition [3].

Incidence rate of occupational injury is the highest in food and drink processing industry, which make it the most dangerous occupation, among the manufacturing industries [4]. In Bralirwa brewery industry, Republic of Rwanda,86.4%of workers suffered from work related injury annually [5]. In Durban, South Africa, about 22% of brewery industry workers were also encountered work related injury within 6 months of working period [6].

In Ethiopia, the number of industries are increasing drastically due to the country’s favorable policy that supports the growth of small and large scale industries [7]. However, workers who are involved in manufacturing industries including brewery companies, have encountered higher level of workplace accident [8]. Though many reasons can be related to work-related injuries; majority (88%) of the injuries are caused by unsafe working practice [9].There is scant knowledge on the level of safety practice and its determinant factors among brewery workers in Ethiopia.

Brewery workers in Ethiopia have been reported to be exposed to various work hazards (excessive heat and noise levels, broken bottles, chemicals and radiation) [7]. This may end up with occupational injuries and diseases, like skin cuts and lacerations, eye injuries, respiratory problems (bronchitis and asthma), hearing impairment, skin diseases, and musculoskeletal disorder [6, 10, 11].

## Methods

### Aim of this study

The aim of this study was to assess the level of self-reported safety practices and associated factors among workers in Dashen Brewery Share Company.

### Study design

Institution based cross-sectional study.

### Study area and period

Dashen Brewery Share Company is situated in the Gondar town, approximately 750 km away from Addis Ababa, the capital city of Ethiopia. It was established in 2000 and currently has 858 employees. It has been certified with ISO 9001 quality management system (QMS) and ISO 14001 environmental management system (EMS).

The study carried out from February to March 2016; Permanent and temporary workers who engaged in production process were included in the study.

### Sample size and sampling technique

The sample size was determined using the formula for a single population proportion assuming a margin of error as 5%, expected proportion of safety practice as 58% [12], 95% confidence interval and 10% of non-respondent rate to came up with a sample size of 415 respondents.

We took a list of all production process workersfrom HumanResource department and used it as sampling frame.

The following procedures were followed to identify respondents from the sampling frame;

- First, Stratified sampling was employed assuming that workers in different department would exhibit different level of safety practices. The calculated sample size was allocated to each stratum proportionally to the sample size.
- Secondly, systematic random sampling was used to select respondent from each stratumand came up with a desired sample size of 415 fromthe following; 22 respondents from Quality department, 195 from Packaging department, 107 from Engineering department, 29 from Brewing department, and 62 from Loading and unloading function.

### Operational definitions

- **Safety Practice:** Respondent’s score out of 20 questions was graded as Good if ≥60%; and poor <60% [13].
- **Attitude:** Attitude of the participants regarding occupational safety and health calculated from 5 point Likert scale questions.Each question had a value of 1–5 that corresponds with the scale measurement. The participants’ answer graded as good attitude if the cumulative answer is ≥80% (20–25), medium attitude if 60–79% (15–19) and poor attitude if (<59%) 0–14 [14].
- **Knowledge:** Participants asked to answer 9 knowledge questions about safety and health. Graded as having “Good knowledge” if they had answered correctly (≥80%) 7–9 questions, (60–80%)5–6 as “Medium level knowledge” and (<59%) 0–4 as “Poor knowledge” [14].
- **Job satisfaction:** Whether the worker was happy or not with the job that he or she had engaged currently [15].
- **Sleep disturbance problem:** The presence of sleeping problems when the worker was at work in the factory [16].
- **Chat chewers:** Chewing chat leaves by the worker at least once per week [16].
- **Management support:** was measured by 5 point Likert scale (Strongly agree =5, Agree =4, Moderate =3, Disagree =2, and Strongly disagree =1) [17].

### Data collection tools

The data was collected using interview administered questionnaire and observation checklist. The data collection were carried out by six occupational safety and health degree graduates.Two supervisorswere also involved for monitoring data collection and checking the completeness of the questionnaires.

### Data quality control

Training was given for data collectors and supervisors for 3 days on procedures, techniques and ways of collecting the data. Prior to the commencement of the actual data collection process, the questionnaire was pretested on 21 (5% of sample size) Pepsi soft drink industry workers and the necessary modification was made. Reliability test was conducted and the consistence of the research tool was ascertained (Additional file 1). Clear introduction explaining the purpose and objective of the study was provided to the respondents on the first page of the questionnaire before data collection. In addition continuous and strict supervision and on spot checking was carried out during the data collection process.

### Ethical consideration

Before data collection, ethical clearance was obtained from Institutional Review Board (IRB) of University Of Gondar, College of Medicine and Health Sciences. The company manager was communicated through formal letters from University of Gondar, Institute of Public Health and permission was obtained. Written consent was sought from each respondent after explaining the purpose and objectives of the study. Confidentiality was assured for information collected from study participants.Privacy was also ensured during the interview.

### Data processing and analysis

Data was entered using Epi-Info version 7, andanalyzed using SPSS statistical package for windows, version 20.0. All assumptions applied to binary logistic regression including fitness of model were checked. To determine factors associated with self-reported level of safety practice, Binary Logistic Regression model was fitted and variables with a *p*-value <0.2 in bivariable analysis were included in the multi-variant analysis. A *p*-value of less than 5% in the multi-variable analysis wasconsidered statistically significant.Crude adds (COR) ratios and adjusted Odds ratios (AOR) with 95% confidence intervalare reportedin the result.

## Results

A total of 415 (100%) respondents were interviewed. Of those individuals almost three fourth 308 (74.2%) of the participants were male. The majority 250 (60.2%) respondents were aged between 14 and 29 years, with mean (±SD) age of 28.18 (±8.67) years. Nearly half of them 207 (49.9%) were diploma holders. Furthermore, 224 (54.0%) were married and more than half of respondents 255 (69.9%) were permanently employed, of which 244 (58.8%) of the respondents had worked for less than or equal to 5 years (Table 1).

**Table 1:** Distribution of Socio-demographic characteristics of respondents, Dashen Brewery Share Company workers (*N* = 415)

Variables	Number	Percent
Age (years)		
14–29	250	60.2
30+	165	39.8
Sex		
Male	308	74.2
Female	107	25.8
Educational level		
Grade 1–8	32	7.7
Grade 9–12	116	28
Diploma	207	49.9
Degree and above	60	14.5
Marital Status		
Married	224	54
Single	180	43.4
Divorced	8	1.9
Widowed	3	0.7
Work Experience (years)		
≤ 5	244	58.8
> 5	171	41.2
Employment Status		
Temporary	125	30.1
Permanent	290	69.9
Working Department		
Quality	22	5.3
Engineering	107	25.8
Packaging	195	47
Brewing	29	7
Loading/Unloading	62	14.9

The current study showed that majority 362 (87.2%) of the workers reportedhaving good level of safety practices.

This study depicted that majority of workers 357 (86%) had utilized at least one type of personal protective equipment (PPE), while were working. For those who didn’t use PPE, lack of PPE 155 (37.3%) and the feeling discomfort while using PPE 65 (15.7%) were the major reasons. The adherence level of safety procedures reported by the workers and their actual performance ascertained by observation showed discrepancy, but it was not statistically significant (*P* = 0.082) (Table 2).

**Table 2:** Use of utilization of safety methods ascertained by self-report from interviewees and by researcher’s observation in Dashen Brewery Share Company. The difference rated by observation and self-report were tested by Mann-Whitney test and the result shows the difference was not significant (*p* = 0.082)

Safety Practice	Self-reported safety practices		Observed safety practices	
	Number	Percent (%)	Number	Percent (%)
Safety shoes	349	84.1	320	77.1
High visibility vest	348	83.9	314	75.7
Overall	341	82.2	310	74.7
Goggles	324	78.1	210	50.6
Gloves	294	70.8	205	49.4
Ear plugs	299	72	179	43.1
Helmet	42	10.1	4	1
Respirator	235	56.6	0	0
Followed the correct manual lifting techniques	178	42.9	149	35.9
Followed demarcated safe walk ways	313	75.4	259	62.4

Multivariate analysis showed that socio-demographic factors; age, marital status, and employment status were found to be the determinant factors for safety practices. Similarly factors like, attitude towards safety practice, management support and training on safety and health were found to be determinant factors for having good safety practice in the study (Table 3).

**Table 3:** Multivariable analysis of predictors for self-reported safety practice in Dashen Brewery Share Company workers (*N* = 415)

Variables	Safety Practice		Crude OR (95%CI)	Adjusted OR (95%CI)
	Good n (%)	Poor n (%)		
Age (years)				
14–29	229 (91.6)	21 (8.4)	2.6 (1.5,4.7)*	7.2 (1.9,26)**
30+	133 (80.6)	32 (19.4)	1.0	1.00
Sex				
Male	274 (89)	34 (11)	1.7 (0.9,3.2)	
Female	88 (82.2)	19 (17.8)	1.0	
Educational level				
Grade 1–8	19 (59.4)	13 (40.6)	1.0	
Grade 9–12	86 (74.1)	30 (25.9)	2.0 (0.86,4.44)	
Diploma	198 (95.7)	9 (4.3)	15.0 (5.6,39.7)	
Degree and above	59 (98.3)	1 (1.7)	40.3 (4.9329)	
Marital status				
Married	212 (94.6)	12 (5.4)	1.0	1.0
Single	140 (77.8)	40 (22.2)	0.2 (0.1,0.39)*	0.1 (0.04,0.42)**
Working department				
Quality	20 (90.9)	2 (9.1)	1.0	
Engineering	102 (95.3)	5 (4.7)	2.0 (0.37,11.3)	
Packaging	176 (90.3)	19 (9.7)	0.9 (0.2,4.3)	
Brewing	26 (89.3)	3 (10.3)	0.9 (0.13,5.7)	
Loading/Unloading	38 (61.3)	24 (38.7)	0.2 (0.03,0.74)	
Employment Status				
Temporary	84 (67.2)	41 (32.8)	1.0	1.0
Permanent	278 (95.9)	12 (4.1)	11.3 (5.7,22.5)*	5.4 (1.3,21.54)**
Knowledge				
Good	285 (90.5)	30 (9.5)	4.0 (1.5,11.4)*	
Medium	63 (78.8)	17 (21.5)	1.6 (0.5,4.8)	
Poor	14 (70)	6 (30)	1.0	
Attitude				
Good	209 (96.8)	7 (3.2)	33.6 (13.9,81.8)*	20.3 (5.8,71.05)**
Medium	121 (92.4)	10 (7.6)	13.6 (6.1,30.3)*	10.7 (3.2,35.9)**
Poor	32 (47.1)	36 (52.9)	1.0	1.0
Job satisfaction				
Yes	216 (89.3)	26 (10.7)	1.5 (0.86,2.74)	
No	146 (84.4)	27 (15.6)	1.0	
Management support				
Yes	252 (96.6)	9 (3.4)	11.0 (5.3,23.7)*	12.0 (3.4,41.9)**
No	110 (71.4)	44 (28.6)	1.0	1.0
Safety and health Training				
Yes	228 (96.6)	8 (3.4)	9.6 (4.4,20.9)*	4.5 (1.2,16.3)**
No	134 (74.9)	45 (25.1)	1.0	1.0

Note: 1:0 = Reference, * Significant at *P* value <0.02, ** Significant at *P* value <0.05

Workers aged14–29 years were 7.2 times more likely to reporthaving good safety practice than older workers[AOR; 7.2;95%; CI; (1.9–26)]. Marital status of workers was also found to be associated with safety practice. Single workers were 86% less likely to reporthaving good safety practice than married workers [AOR; 0.1; 95%; CI; (0.04–0.4)].

Employment status showed a statistically significant association with safety practice; in which permanent workers 5.35 times more likely to reportgood safety practice than their counterparts[AOR; 5.4; 95%; CI; (1.3–21.5)].

Workers who reported good attitude towards safety practice were 20.3 times more likely to report good safety practice than employees who reporting poor attitude [AOR; 20.3; 95%; CI; (5.8–71.1)]. The study also revealed that workers who perceived they had support by management were 11.9 times more likely to report good safety practice than workers who perceived they didn’t get management support [AOR; 12.0;95%; CI; (3.4–41.9). On the other hand, those workers who attended safety and health training were 4.5 times more likely to reporthaving good safety practice than workers who didn’t get safety and health training [AOR; 4.5; 95%; CI; (1.2–16.3)] (Table 3).

## Discussion

The level of self-reported safety practices in Dashen Brewery was very good; in which 87.2% of the workers reported to comply with good level of safety practices. The result of this study is in agreement with the study conducted among pipeline workers in Nigeria that report 85.9% of the workers having good safety practice [13]. On the other hand, the level of self-reported safety practice was relatively good compared to other studies conducted in Ethiopia among laboratory workers 39.3% [18], in Iran among chemical industry workers 70% [19], and also in Iran among steel manufacturing workers 58.2% [20].The reason for high safety practice by workers might be related to the adoption of principles of QMS and EMS by the company. Activities undertaken to qualify for these systems might have a positive effect on safety practice of the workers. Hence, our findings may support that implementing QMS andEMS to improvesafety and health is very crucial [21, 22].

Though not statistically significant, the discrepancy on the level of adherence to safety procedures based on self-report by the workers and actual performance ascertained by observation might be due to social desirability bias. The bias could be apparent if less robust data collection tools and techniques are used during face to face interview. Hence, adopting robust data collection tools and techniques are very important to minimize the bias [23]. In the current study, we have used pre-tested, validated and robust tools for data collection to overcome the problem.

This study found that younger workers were more likely to report good safety practices than old workers. This finding is in agreement with a study from India [24].The possible explanation could be younger workers show better motivation and information about safety and health as shown in our result. However, other studies conducted in Ethiopia found that younger workers were more likely to suffer from occupational injury thanolder workers [7, 12, 16]. In general as employees grow older, the behavior of workers improved [19, 20, 25, 26].

In our study, marital status was also significantly associated with safety practices.Married workers were more likely to report good safety practice than singles. This finding is consistent with study conducted in Komobolcha textile factory, Ethiopia [15]. One possible reason for this might be thatmarried workers wanted to take care of themselves due to their concerns for being able to provide for their partners and families [15].

Employment status was also found to be a significant predictor of safety practice; permanent workers being more likely to report good safety practice than temporary workers. This finding was consistent with another study conducted in Ethiopia [27]. The possible explanation for this could be the difference in benefit packages, the company provides to permanent and temporary workers. Hence, temporary workers have limited access to basic safety training and use of personal protective devices. In another study conducted in Spain, the authors reasoned that job dissatisfaction and less knowledge and experience of the workplace were associated with high prevalence of occupational injury among temporary workers [28].

In the current study, attitude were associated with safety practice. This finding is in accordance corroborate with a study conducted among chemical industry workers in Iran [19]. Moreover, Another Iranian study in gas refineries reported a decrease in number of accidents with increasing safety attitudes [29]. The possible reason for this might be the existence of a direct relationship between good attitude toward safety and the actual safety practice among workers.

In this study, management support was a significant determinant factor for safety. The findings was congruent with studies conducted across the globe [30–33]. The result of this study is also in line with the view of the British Health and Safety Executive (HSE) [33]. The reason for this finding might be due to the fact that employees behave according to good safety practices when they perceive that the management value them and cares for their personal well-being.

In the present study, respondents who got safety and health training were more likely to report good safety practice than their counterparts. The result was also like the findings of a study conducted in Tendaho, Ethiopia [27]. Safety training was also indicated as important barrier for work-related injuries in different industries [10, 34]. This is based on the idea that training make employees aware of possible dangers and ways to avoid occupational injury.

## Conclusion

The reported level of safety practice in this study was good. Management commitment to safety is an important factor for safety of industry workers. Training of new employees as well as temporary and permanent workers about safety and health specifically on the nature of the work place, hazard prevention and control methods is very important and should be provided regularly. Well-designed work procedures, which are fitting to the specific working conditions, should be readily available to improve adherence of hazard preventive measures, like PPE.

## Additional files

Additional file 1: Reliability and Hosmer Lemeshow test. (DOC 21 kb)

## Abbreviations

EMS: Environmental Management System
HSE: Health and Safety Executive
ILO: International Labor Organization
IRB: Institutional Review Board
ISO: International Standard Organization
PPE: Personal Protective Equipment
QMS: Quality Management System
SPSS: Statistical Package for Social Sciences.
