# Supplementary material for: Self-reported safety practices and associated factors among employees of Dashen brewery share company, Gondar, Ethiopia: a cross-sectional study
Source: J Occup Med Toxicol. 2017 Aug 4;12:22. doi: 10.1186/s12995-017-0169-2 (PMC5544972; doi:10.1186/s12995-017-0169-2)
Supplement: Supplementary file 1 — Reliability and Hosmer Lemeshow test. (DOC 21 kb) [file 12995_2017_169_MOESM1_ESM.doc]

**Annex I**

**Consent Form**

University of Gondar

College of Medicine and health sciences

Institute of Public Health

Department of Environmental and Occupational Safety and Health

Survey questionnaire to assess safety practice and associated factors among employees of Dashen BreweryShare Company in Gondar City, Amhara Region, North West of Ethiopia,

My name is --------------------------. I am working in the research team of University of Gondar college of Medicine and health sciences institute of public health Department of Environmental and Occupational Safety and Health. I would like to ask you a few questions about safetypractice. This will help us to improve occupational safety, health and working environment services provided to you. Your name will not be written in this form and will never be used in connection with any information you tell us. All information given by you will be kept strictly confidential. Your participation is voluntary and you are not obliged to answer any question you do not wish to answer. If you fill discomfort with the interview please fill free to drop it at any time. This interview will take about 30 minutes. Do I have your permission to continue?

1. If yes, continue to the next page

2. If no, skip to the next participant by writing reasons for his/ her refusal

Name and signature of the interviewer who sought the consent_________________

Date of interview ______/ /________

Date/ Month /Year

Result of interview: 1.Completed 2.Respondent not available 3.Refused4. Partially completed

Checked by Supervisor: Name____________Sig._________ Date_________

**English version information sheet**

**Name of the investigator:** Solomon Tesfa, Daniel Haile, and Awrajaw Dessie

**Name of organization:** University of Gondar, college of Medicine and Health science Institute of Public Health.

**Title of the study:**Safety practices and associated factors among employees of Dashen BreweryShare Company, Gondar town, North West Ethiopia.

**Objective of the study:** Assessment of safety practices and associated factors among employees of Dashen BreweryShare Company, Gondar town, North West Ethiopia.

**Introduction:** This information sheet and consent form is prepared to explain the study you are being asked to join. Please listen carefully and ask any questions about the study before you are agree to join. You may ask questions at any time after joining the study.

**Procedure:** To assess level of safety practice and associated factors among employees of Dashen brewery Share Company, we invite you to take part in this study. If you are willing to participate in this study, you need to understand and sign the agreement form. Then after, you will be interviewed by the data collector to give your response. You do not need to tell your name to the data collector and all your response will be kept confidentially by using coding system whereby no one will have access to your response.

**Risk of the study:** The study has no any risk for the participant and interview also will be private to make safe participants from management related problems.

**Benefit of the study:** The study participants will not get direct benefit for being participated. The result will be used as a baseline for further studies that can be done in these areas. The result will be presented to Ministry of labor and social affair, Amhara National Regional State Labor and social affair office, North Gondar Labor and social affair office and for Dashen Brewery Share Company for designing and implement occupational safety andhealth enhancing program at work place.

**Confidentiality:** The information collected from this study will be kept confidential and information about you that will be collected by this study will be stored in a file, without your name, but a code number assigned to it and it will not be revealed to anyone except the principal investigators.

**Right to refuse or withdraw:** you have full right to refuse from participating in this research. You can choose not to respond to some or all questions. You have also the full right to withdraw from this study at any time you wish.

**Persons to contact:**

1. Solomon Tesfa

Email: soloteshi@gmail.com

2. Daniel Haile

Email:daniel.haile7@gmail.com

3. Awrajaw Dessie

Email: awrajawdss@gmail.com

Questionnaire identification number_______________

**Part-1:** Socio-demographic characteristics of participants.

| SN | Question | Possible answer | Code | Skip |
| --- | --- | --- | --- | --- |
| 101 | Age | 1. 14-29 2. 30-44 3. 45+ |  |  |
| 102 | Sex | 1. Male 2. Female |  |  |
| 103 | Educational level | 1. Illiterate 2. Read and Write 3. Grade 1-8 4. Grade 9-12 5. Diploma level 6. Degree & above level |  |  |
| 104 | Marital status | 1. Married 2. Single 3. Divorced 4. Widowed |  |  |
| 105 | Work experience | ------------------------ years |  |  |
| 106 | Employment status | 1. Temporary worker 2. Permanent worker |  |  |
| 107 | Working Department | …………………..department |  |  |

**Part-2**: Behavioral characteristics of Participants.

| SN | Question | Possible answer | | | Code | Skip |
| --- | --- | --- | --- | --- | --- | --- |
| 201 | Do you chew chat? | 1. Yes 2. No | | |  | If no Q201skip to Q203 |
| 202 | If yes to Q203, how often? | 1. Every day 2. 1-3 days/week 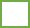 3. Occasionally 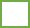 | | |  |  |
| 203 | Knowledge of workers on safety |  | | |  |  |
| 203.1 | Do you aware about occupational health and safety policy of your organization? | 1. Yes 2. No | | |  |  |
| 203.2 | First Aid kits are available for the emergency conditions in workplace. | 1. True 2. False | | |  |  |
| 203.3 | Workplace hazards such as noise or chemicals can cause a long or short term detrimental health effects? | 1. True 2. False | | |  |  |
| 203.4 | Keeping the workplace clean is keeping safe? | 1. True 2. False | | |  |  |
| 203.5 | Utilization Personal protective equipment is one of the hazard control measure? | 1. True 2. False | | |  |  |
| 203.6 | Do you aware of occupational hazards exist in your workplace? | 1. Yes 2. No | | |  |  |
| 203.7 | Do you know how fire extinguisher implement? | 1. Yes  2. No | | |  |  |
| 203.8 | Do you know any occupational health and safety information that can be described in pictures, signs, labels or other else? | 1. Yes 2. No | | |  |  |
| 203.9 | Are you aware of safety measures to protect you from workplace hazards? | 1. Yes 2. No | | |  |  |
| 204 | Regarding Attitude towards safety. | | | | | |
|  |  | Strongly disagree | disagree | Neutral | Agree | Strongly agree |
| 204.1 | Do you think provided safety instruction is relevant? |  |  |  |  |  |
| 204.2 | Do you think as you are at risk of acquiring illness and injury due to hazards at the workplace? |  |  |  |  |  |
| 204.3 | Do you think PPE protects you from hazards? |  |  |  |  |  |
| 204.4 | Do you think Periodic medical examination is necessary? |  |  |  |  |  |
| 204.5 | Hazard control measures are adequate? |  |  |  |  |  |
| 205 | Do you sleep well? Is your sleeping continuous without any intermittence? | 1.Yes 2.No | | |  |  |
| 206 | Are you satisfied with the job you are engaged now? | 1.Yes 2.No | | |  |  |

**Part-3:** Environmental factors for safety practice.

| SN | Question | Possible answer | | | Code | Skip |
| --- | --- | --- | --- | --- | --- | --- |
| 301 | Hours worked per week | ------------------- | | |  |  |
| 302 | Are you trained in Safety and Health issues in your workplace? | 1. Yes 2.No | | |  |  |
| 303 | Have necessary PPE been made available for You? | 1. Yes 2.No | | |  |  |
| 304 | If yes What type of PPE |  | | |  | If no skip Q 305 |
| 304.1 | Gloves | 1. Yes 2.No | | |  |  |
| 304.2 | Ear plug | 1. Yes 2.No | | |  |  |
| 304.3 | Respirators | 1. Yes 2.No | | |  |  |
| 304.4 | Helmet | 1. Yes 2.No | | |  |  |
| 304.5 | Overalls | 1. Yes 2.No | | |  |  |
| 304.6 | Goggles /Safety glasses | 1. Yes 2.No | | |  |  |
| 304.7 | Safety shoe | 1. Yes 2.No | | |  |  |
| 304.8 | High visibility Jacket | 1. Yes 2.No | | |  |  |
|  | Regarding management support |  | | |  |  |
| 305. | Management visibly demonstrates support for employee safety. | 1. Yes 2.No | | |  |  |
|  |  | Strongly disagree | disagree | Moderate | Agree | Strongly agree |
| 306 | The management always provide health and safety tools and equipment |  |  |  |  |  |
| 307 | Respond quickly employees’ safety questions |  |  |  |  |  |
| 308 | Rewarding workers who perform safe |  |  |  |  |  |
| 309 | Conduct periodic follow up |  |  |  |  |  |
| 310 | Investigates safety problems quickly |  |  |  |  |  |
| 311 | Provides safe environmental working condition |  |  |  |  |  |

**Part-4** Question for assessing workers safety practices.

| 401 | Do you use personal protective equipment all time? | 1. Yes 2.No |  | If no skip to Q 401.9 |
| --- | --- | --- | --- | --- |
| 401 | If yes to Q1, What type? |  |  |  |
| 401.1 | Gloves | 1. Yes 2.No |  |  |
| 401.2 | Ear plug | 1. Yes 2.No |  |  |
| 401.3 | Respirators | 1. Yes 2.No |  |  |
| 401.4 | Helmet | 1. Yes 2.No |  |  |
| 401.5 | Overalls | 1. Yes 2.No |  |  |
| 401.6 | Goggles/Safety glasses | 1. Yes 2.No |  |  |
| 401.7 | Safety shoes | 1. Yes 2.No |  |  |
| 401.8 | High visibility Jacket | 1. Yes 2.No |  |  |
| 401.9 | What are the reasons for not using personal protective? | 1. Lack of protective equipment 2. Lack of safety and health education 3. Not comfortable to use 4. Decrease work performance 5. Create safety and health hazards 6. No stringent follow up |  |  |
| 402 | Do you properly set your PPE after your finish your work? | 1. Yes 2.No |  |  |
| 403 | Do you take safety shower after finish your work? | 1. Yes 2.No |  |  |
| 404 | Do you keep cleanness of your working station and tools? | 1. Yes 2.No |  |  |
| 405 | Do you follow stated work procedures when you doing work? | 1. Yes 2.No |  |  |
| 406 | Do you report dangerous situations when you see them in work place? | 1. Yes 2.No |  |  |
| 407 | Have you ever make suggestions to your supervisor for improving health and safety? | 1. Yes 2.No |  |  |
| 408 | Have you ever reported to your supervisor faults or conditions involving a risk for you or other workers? | 1. Yes 2.No |  |  |
| 409 | Have you ever warned other workers about health and safety risks? | 1. Yes 2.No |  |  |
| 410 | Do you report all work-related injuries and illnesses to your supervisor promptly, regardless of how minor they may seem | 1. Yes 2.No |  |  |
| 411 | Do Report any equipment/machine malfunctions or defects to the supervisor? | 1. Yes 2.No |  |  |
| 412 | Do Comply with periodic medical examination? | 1. Yes 2.No |  |  |
| 413 | Do you follow demarcated safety walk ways? | 1. Yes 2.No |  |  |
| 414 | Have you ever asked for personal protection equipment? | 1. Yes 2.No |  |  |
| 415 | Have you ever asked for information regarding health and safety at your workplace? | 1. Yes 2.No |  |  |
| 416 | Obey appropriately for posted safety signage. | 1. Yes 2.No |  |  |
| 417 | Do you follow the correct manual handling techniques (using legs, back straight, weight close to body, feet flat on floor, knees bent)? | 1. Yes 2.No |  |  |
| 418 | Do you share your occupational health and safety knowledge with other new staff members at the workplace? | 1. Yes 2.No |  |  |
| 419 | Do you work with the machines/Equipment’s that you trained for? | 1. Yes 2.No  3. Not applicable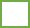 |  |  |

English version Observation Checklist

| S.N | Questions | Yes | No | Remarks |
| --- | --- | --- | --- | --- |
| 1 | Workers wearing proper eye protection for the task (glasses, goggles)? |  |  |  |
| 2 | Workers use proper gloves? |  |  |  |
| 3 | Workers wear proper protective clothing? |  |  |  |
| 4 | Workers wear proper foot protection (safety shoes, boots)? |  |  |  |
| 5 | Workers wearing proper hearing protection where the hazard exists (ear plugs, ear muffs)? |  |  |  |
| 6 | Workers wearing proper Head Protection where hazard exists? |  |  |  |
| 7 | Workers wearing high visibility Jacket when needed? |  |  |  |
| 8 | Workers applies proper manual lifting techniques (using legs, back straight, weight close to body, feet flat on floor, knees bent)? |  |  |  |
| 9 | Workers following demarcated walkways? |  |  |  |
